# Confocal findings of an intradermal nevus in a unique anatomical location: A diagnostic pitfall and histopathologic correlation

**DOI:** 10.1016/j.jdcr.2023.08.014

**Published:** 2023-08-25

**Authors:** Doniya Milani, Katharine Hanlon, Lilia Correa-Selm, James M. Grichnik, Wei-Shen Chen

**Affiliations:** a University of South Florida Morsani College of Medicine, Tampa, Florida; b Department of Dermatology and Cutaneous Surgery, University of South Florida, Tampa, Florida; c Department of Cutaneous Oncology, H. Lee Moffitt Cancer Center, Tampa, Florida

**Keywords:** benign nevi, benign nevus, confocal microscopy, congenital nevi, congenital nevus, dendritic cells, dysplastic nevi, dysplastic nevus, genital nevi, genital nevus, intradermal melanocytic nevi, intradermal melanocytic nevus, intradermal nevi, intradermal nevus, junctional melanocytic hyperplasia, melanocytes, melanoma, melanoma in situ, reflectance confocal microscopy

## Clinical presentation

**Fig 1:**
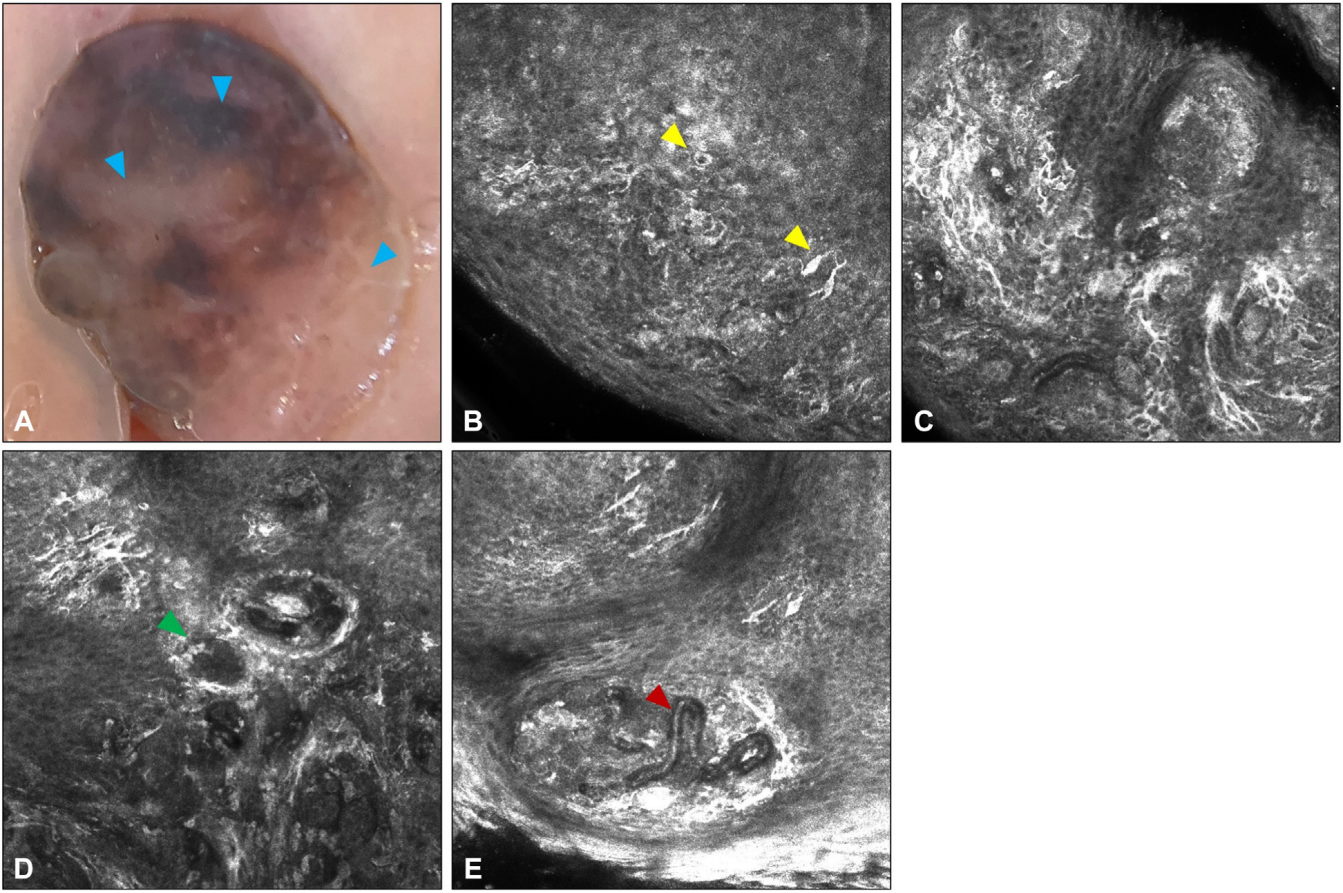
Confocal and dermoscopic images of congenital intradermal melanocytic nevus located on the labia. **A,** Areas of *light to dark brown* structureless pigmentation, patchy hypopigmentation, and a subtle focus of a negative network pattern (*blue arrowheads*). **B,** Pleomorphic dendritic and round nucleated melanocytes high in the epidermis (*yellow arrowheads*). **C,** Prominent junctional melanocytic hyperplasia with irregularly distributed, hyper-refractile dendritic cells approaching confluence in some areas. **D,** Partially-edged dermal papilla (*green arrowhead*) intermixed with dermal nesting. **E,** Distinctive vasculature within the papillary dermis showing a hairpin/serpentine vessel (*red arrowhead*).

A 38-year-old woman presented to our dermatology clinic with a 3-mm, brown papule on the labia minora. Although previously asymptomatic, the patient reported recent irritation. Dermoscopy revealed a heterogeneous pattern with areas of structureless hyperpigmentation, patchy hypopigmentation, and a subtle focus of a negative network pattern (Fig 1, *A*).

## Confocal microscopy appearance

The lesion was further evaluated using reflectance confocal microscopy (RCM). RCM revealed large nucleated cells situated in the midepidermis (Fig 1, *B*), mimicking pagetoid melanocytosis. Dendritic cells with a high reflective signal were seen at the dermoepidermal junction, along with nests, prominent vessels, and partially edged and nonedged dermal papillae (Fig 1, *C-E*). To rule out melanoma in situ, a biopsy was performed.

## Histologic diagnosis

**Fig 2:**
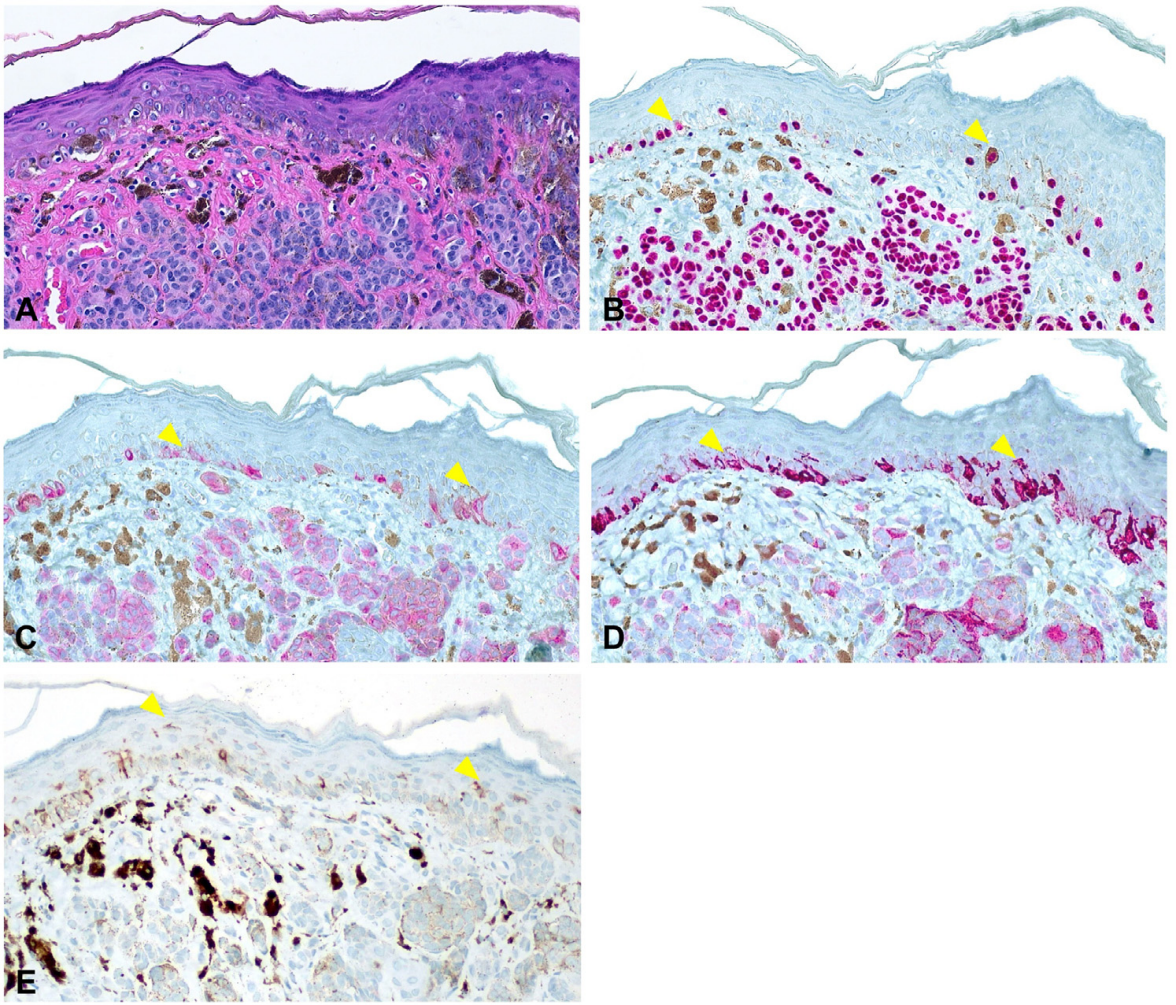
Biopsy specimen revealing a junctional melanocytic hyperplasia overlying a banal intradermal melanocytic nevus. **A,** hematoxylin and eosin–stained section showing nests of benign intradermal melanocytes. **B,** SOX10 immunostains reveals a benign appearing junctional melanocytic hyperplasia (*arrowheads*). Few cells exhibit a suprabasal location. **C** and **D,** Mart1 and HMB45 reveal a highly arborizing and dendritic morphology of these junctional melanocytes (*arrowheads*). **E,** CD1a immunostain reveals dendritic cells in the deep and superficial epidermis (*arrowheads*), morphologically consistent with Langerhans cells.

Histological examination of hematoxylin and eosin–stained sections revealed an intradermal melanocytic nevus (IDN) with congenital features (Fig 2, *A*), a benign lesion requiring no treatment. To correlate the histomorphology with RCM findings, immunohistochemical studies were performed. Mart1 and HMB45 revealed benign intradermal melanocytes and junctional melanocytes with arborizing dendrites (Fig 2, *C* and *D*). A CD1a immunostain was performed to investigate superficial cells observed on RCM (Fig 1, *B*) and revealed dendritic cells scattered within the deep and superficial levels of the epidermis (Fig 2, *E*), morphologically consistent with Langerhans cells.

## Discussion

RCM is a noninvasive skin imaging technique that provides a superficial and horizontal observational window. Frequently employed as a supplementary tool to dermoscopy, accurate RCM interpretation requires knowledge of diagnostic pitfalls. Typical RCM features for IDN include a honeycomb pattern of keratinocytes, edged papillae, and comma vessels.^1^ Expected confocal findings for melanoma include atypical dendritic cells in the upper epidermis, dermoepidermal junction disarray, and irregular nests of melanocytes.^2^ Our case demonstrated large clods on RCM, consistent with IDN; however, the partially edged papillae, prominent hairpin vessels, and pagetoid dendritic cells raised concern for melanoma.

To explain the discordance between the concerning RCM findings and the benign histopathologic results, we first hypothesized that the observed dendritic cells represented junctional melanocytic hyperplasia overlying an IDN. This phenomenon, which is associated with melanoma mimicry on superficial biopsies, was first reported by Okamura et al^3^ and later by Massi and LeBoit.^4^ Though initial immunostaining revealed a lentiginous distribution of junctional melanocyte hyperplasia with suprabasal migration, these melanocytes do not entirely account for the superficial cells seen on RCM. As Langerhans cells have been shown to generate reflection indices and dendritic morphology resembling melanocytes,^5^ we performed CD1a immunostains and confirmed their abundant presence within the epidermis.

Melanocytic vulvar lesions are diagnostically challenging for several reasons. One such factor is the special site nevus phenomenon, where melanocytic nevi in certain locations share features with dysplastic nevi. This complexity is confounded by the possibility of overlying junctional melanocytic hyperplasia and the abundance of Langerhans cells within the mucosa. Analogously, these challenges are mirrored when employing RCM.

Given these diagnostic challenges, caution and prudence remain critical when evaluating pigmented lesions on mucosal sites with RCM. As RCM gains clinical prominence, patterns previously described in histopathology will be rediscovered. By examining lesions from various dimensional perspectives, RCM and histomorphology can mutually benefit from each other’s advancement.

## Conflicts of interest

Dr Correa-Selm is a consultant for Accutec and a consultant and researcher for Novartis Pharmaceutical, also serves on the Advisory Board for the Jacinto Convit World Organization and the Dermatology Advisory for Melanoma Research Foundation. Dr Grichnik is a consultant for Galileo Group and Canfield Scientific; serves on the Skin Advisory Board for Regeneron and the Dermatology Advisory for Melanoma Research Foundation; and receives clinical trial support from Novartis, Eli Lilly, Dermira, Elorac, Boehringer, and Amgen. Author Milani, Author Hanlon, and Dr Chen have no conflicts of interest to declare.

## References

[1] Shahriari N., Grant-Kels J.M., Rabinovitz H., Oliviero M., Scope A. (2018). In vivo reflectance confocal microscopy image interpretation for the dermatopathologist. J Cutan Pathol.

[2] Agozzino M., Moscarella E., Babino G., Caccavale S., Piccolo V., Argenziano G. (2019). The use of in vivo reflectance confocal microscopy for the diagnosis of melanoma. Expert Rev Anticancer Ther.

[3] Okamura J.M., Barr R.J., Cantos K.A. (2000). Benign atypical junctional melanocytic hyperplasia associated with intradermal nevi: a common finding that may be confused with melanoma in situ. Mod Pathol.

[4] Massi G., LeBoit P.E. (2016).

[5] Hashemi P., Pulitzer M.P., Scope A., Kovalyshyn I., Halpern A.C., Marghoob A.A. (2012). Langerhans cells and melanocytes share similar morphologic features under in vivo reflectance confocal microscopy: a challenge for melanoma diagnosis. J Am Acad Dermatol.

